# Sarcopenia and Sarcopenic Obesity and Mortality Among Older People

**DOI:** 10.1001/jamanetworkopen.2024.3604

**Published:** 2024-03-25

**Authors:** Elizabeth Benz, Alexandre Pinel, Christelle Guillet, Frederic Capel, Bruno Pereira, Marie De Antonio, Melanie Pouget, Alfonso J. Cruz-Jentoft, Doris Eglseer, Eva Topinkova, Rocco Barazzoni, Fernando Rivadeneira, M. Arfan Ikram, Marinka Steur, Trudy Voortman, Josje D. Schoufour, Peter J.M. Weijs, Yves Boirie

**Affiliations:** 1 Human Nutrition Unit, Clermont Auvergne University, Institut National de Recherche pour l’Agriculture, l’Alimentation et l’Environnement, Centre de Recherche en Nutrition Humaine, Clermont-Ferrand, France; 2 Department of Epidemiology, Erasmus University Medical Center, Rotterdam, the Netherlands; 3 Department of Internal Medicine, Erasmus University Medical Center, Rotterdam, the Netherlands; 4 Unit of Biostatistics, Clermont-Ferrand University Hospital, Clermont-Ferrand, France; 5 Department of Clinical Nutrition, Clermont-Ferrand University Hospital, Clermont-Ferrand, France; 6 Servicio de Geriatria, Hospital Universitario Ramon y Cajal (IRYCIS), Madrid, Spain; 7 Institute of Nursing Science, Medical University of Graz, Graz, Austria; 8 Department of Geriatrics, First Faculty of Medicine, Charles University and General University Hospital, Prague, Czech Republic; 9 Department of Medical, Technological and Translational Sciences, University of Trieste, Ospedale di Cattinara, Trieste, Italy; 10 Faculty of Sports and Nutrition, Centre of Expertise Urban Vitality, Amsterdam University of Applied Sciences, Amsterdam, the Netherlands; 11 Department of Nutrition and Dietetics, Amsterdam University Medical Centers, Amsterdam Public Health Institute, Vrije Universiteit, Amsterdam, the Netherlands

## Abstract

**Importance:**

Sarcopenia and obesity are 2 global concerns associated with adverse health outcomes in older people. Evidence on the population-based prevalence of the combination of sarcopenia with obesity (sarcopenic obesity [SO]) and its association with mortality are still limited.

**Objective:**

To investigate the prevalence of sarcopenia and SO and their association with all-cause mortality.

**Design, Setting, and Participants:**

This large-scale, population-based cohort study assessed participants from the Rotterdam Study from March 1, 2009, to June 1, 2014. Associations of sarcopenia and SO with all-cause mortality were studied using Kaplan-Meier curves, Cox proportional hazards regression, and accelerated failure time models fitted for sex, age, and body mass index (BMI). Data analysis was performed from January 1 to April 1, 2023.

**Exposures:**

The prevalence of sarcopenia and SO, measured based on handgrip strength and body composition (BC) (dual-energy x-ray absorptiometry) as recommended by current consensus criteria, with probable sarcopenia defined as having low handgrip strength and confirmed sarcopenia and SO defined as altered BC (high fat percentage and/or low appendicular skeletal muscle index) in addition to low handgrip strength.

**Main Outcome and Measure:**

The primary outcome was all-cause mortality, collected using linked mortality data from general practitioners and the central municipal records, until October 2022.

**Results:**

In the total population of 5888 participants (mean [SD] age, 69.5 [9.1] years; mean [SD] BMI, 27.5 [4.3]; 3343 [56.8%] female), 653 (11.1%; 95% CI, 10.3%-11.9%) had probable sarcopenia and 127 (2.2%; 95% CI, 1.8%-2.6%) had confirmed sarcopenia. Sarcopenic obesity with 1 altered component of BC was present in 295 participants (5.0%; 95% CI, 4.4%-5.6%) and with 2 altered components in 44 participants (0.8%; 95% CI, 0.6%-1.0%). An increased risk of all-cause mortality was observed in participants with probable sarcopenia (hazard ratio [HR], 1.29; 95% CI, 1.14-1.47) and confirmed sarcopenia (HR, 1.93; 95% CI, 1.53-2.43). Participants with SO plus 1 altered component of BC (HR, 1.94; 95% CI, 1.60-2.33]) or 2 altered components of BC (HR, 2.84; 95% CI, 1.97-4.11) had a higher risk of mortality than those without SO. Similar results for SO were obtained for participants with a BMI of 27 or greater.

**Conclusions and Relevance:**

In this study, sarcopenia and SO were found to be prevalent phenotypes in older people and were associated with all-cause mortality. Additional alterations of BC amplified this risk independently of age, sex, and BMI. The use of low muscle strength as a first step of both diagnoses may allow for early identification of individuals at risk for premature mortality.

## Key Points

**Question:**

What is the prevalence of sarcopenia and sarcopenic obesity, and are these conditions associated with all-cause mortality?

**Findings:**

In this cohort study of 5888 older adults, 2.2% had confirmed sarcopenia; by applying the sarcopenic obesity algorithm, 5.0% had sarcopenic obesity with 1 and 0.8% with 2 altered components of body composition. The 10-year mortality risk was particularly high for participants with confirmed sarcopenia as well as sarcopenic obesity with 1 or 2 altered components of body composition.

**Meaning:**

These findings suggest that sarcopenic obesity may be associated with worse survival, and conducting screening for muscle function may help prevent premature death among older people.

## Teaser

This cohort study of older adults in the Netherlands examined the associations of sarcopenia and sarcopenic obesity with mortality risk during a 10-year follow-up period.

## Introduction

Age-related body composition (BC) changes are characterized by an increase in fat mass and a steady decrease in both muscle function and mass. These changes lead to different clinical and functional phenotypes, such as sarcopenia and sarcopenic obesity (SO), which contribute to increased morbidity and mortality.^1,2,3,4,5^ As life expectancy has increased, the proportion of older people with high body fat and/or low muscle function and mass has progressively risen.^6,7,8^ Nevertheless, sarcopenia and SO are closely related conditions that are still rarely detected and untreated in clinical practice.^9,10,11^

Sarcopenia, with an estimated prevalence ranging from 3.2% to 26.3%, is characterized by low muscle function and mass.^12,13,14^ Obesity, or high body fat mass, is the most common chronic disease in the world.^8^ Sarcopenia can occur concurrently with, and may be worsened by, body fat gain in the context of obesity, a condition recently defined as SO by the European Society for Clinical Nutrition and Metabolism (ESPEN) and the European Association for the Study of Obesity (EASO).^3^ Previous studies found a global prevalence of SO of 11% (95% CI, 10%-13%) using various definitions, cutoffs, techniques, and population setting.^15^ Using the contemporaneous ESPEN/EASO definition, more recent studies have found a prevalence of SO ranging from 7.9% to 23% in clinical^16^ and 7.1% to 9.6% in community-dwelling settings.^17,18,19^

Obesity and sarcopenia are both independently linked to adverse outcomes,^20,21^ but their combination might act synergistically, amplifying their health-threatening effects.^22,23,24,25,26,27,28,29^ Although the definition of SO is still evolving, it is recognized as a scientific and clinical priority among people at increased risk of cardiometabolic and functional deficiencies.^4,24,30^ Moreover, the prevalence of SO and its association with risk of mortality among the general population remains unknown and needs to be evaluated to understand the potential clinical impact of SO.^31,32^ Therefore, this study examined the prevalence of SO at the population level using the most recent definition and evaluated associations of sarcopenia and SO with mortality risk during a 10-year follow-up period among participants of a large-scale, population-based study.

## Methods

### Study Design and Participants

This cohort study included individuals from the Rotterdam Study, a prospective, population-based cohort study ongoing since 1989.^33^ Briefly, the Rotterdam Study includes almost 15 000 participants 45 years or older living in Ommoord district in the city of Rotterdam, the Netherlands. The ethnicity of the Rotterdam Study represents a homogeneous population largely of European ancestry (98.0%). Every 4 to 5 years, participants undergo follow-up examinations, testing, and monitoring of clinically significant outcomes at the research center. We included all participants who visited the research center between March 1, 2009, and June 1, 2014, and excluded all of those who had no reliable or available measurements of handgrip strength and dual-energy x-ray absorptiometry (DXA) scan. The Rotterdam Study has been approved by the Medical Ethics Committee of Erasmus Medical Center University according to the Population Study Act Rotterdam Study, executed by the Ministry of Health, Welfare, and Sports of the Netherlands. In accordance with the Declaration of Helsinki,^34^ all included participants provided written informed consent to participate in the study and to obtain information from their treating physicians. This study adheres to the Strengthening the Reporting of Observational Studies in Epidemiology (STROBE) reporting guideline.

### Assessment of Obesity

Height and weight were measured at the research center with individuals barefoot in standing position wearing light indoor clothes. Body mass index (BMI) was calculated as weight in kilograms divided by height in meters squared. Obesity was defined based on a BMI of 27 or greater because the median (IQR) BMI of 27 (25-30) reflects a significant correlation (*r* > 0.7, *P* < .001) with an excess of fat percentage in this study population (eFigure 1 in Supplement 1). In addition, previous studies have reported that a BMI of 27 or greater can predict fat percentage in older people.^35^

### Assessment of Sarcopenia

We defined sarcopenia according to the updated European Working Group of Sarcopenia in Older People (EWGSOP2) criteria^12^ (eTable 1 in Supplement 1). Probable sarcopenia was defined as having a low handgrip strength and was confirmed as having a low appendicular skeletal muscle mass index. Maximum handgrip strength was obtained as the maximum value of 3 trials performed in the nondominant hand. Low handgrip strength was defined as less than 27 kg for men and less than 16 kg for women.^36^ The same examinator measured lean mass by DXA scan, using a total body-beam densitometer (iDXA, GE Lunar Corp). The scans were analyzed with Encore software, version 13.6 (Encore Software LLC), providing measurements across predefined body regions of interest, namely, the head, trunk, arms, and legs. The sum of the lean mass from the upper and lower limbs is called appendicular lean mass (ALM), and appendicular skeletal muscle index (ASMI) was defined as ALM divided by height squared. Low ASMI was defined as less than 7.0 for men and less than 5.5 for women.^12^

### Assessment of SO

**Figure 1.**
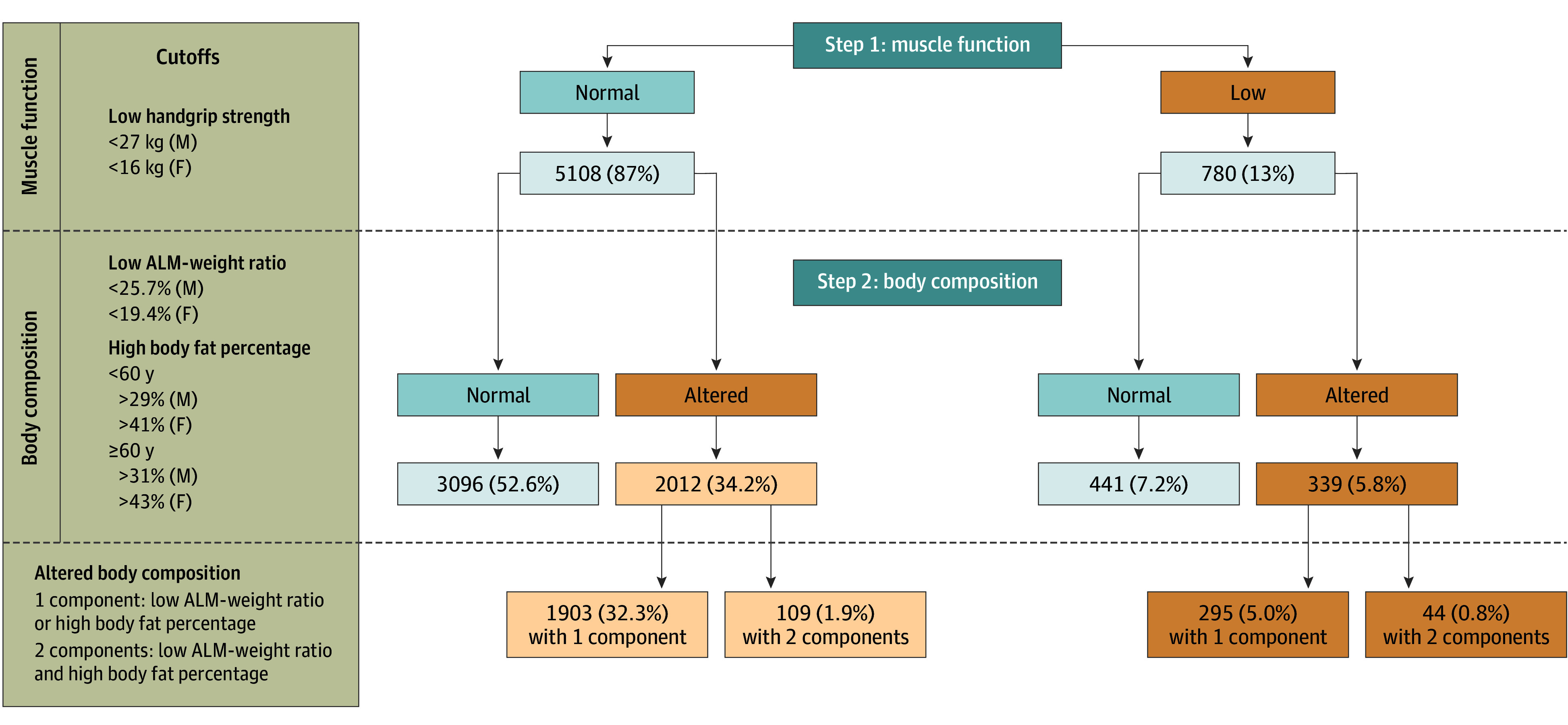
Flowchart of the Study Population Diagnosed as Having Sarcopenic Obesity Diagnostic criteria for sarcopenic obesity were adapted from the European Society for Clinical Nutrition and Metabolism and the European Association for the Study of Obesity consensus. Cutoffs for muscle function were a low handgrip strength of less than 27 kg for men and less than 16 kg for women. Cutoffs for body composition were low appendicular lean mass (ALM) divided by weight of less than 25.7% for men and less than 19.4% for women and high percentage of body fat of more than 29% for men and more than 41% for women at younger than 60 years and greater than 31% for men and greater than 43% for women at 60 years or older. For altered body composition, 1 component indicates low ALM divided by weight or high percentage of body fat and 2 components indicate low ALM divided by weight and high percentage of body fat.

Sarcopenic obesity was defined using the ESPEN/EASO Consensus Statement (eTable 2 in Supplement 1).^3^ We applied the diagnosis criteria of this consensus to all participants. We determined 6 categories for those participants who have normal or low handgrip strength, with or without altered BC. Participants with low handgrip strength and altered BC (1 or 2 components: high fat percentage and/or low ALM-weight ratio) were considered to have SO (Figure 1). In addition, we screened all participants according to a BMI of 27 or greater.

Handgrip strength and ALM were measured as explained earlier. Low lean mass was defined as an ALM-weight ratio of less than 25.7% for men and less than 19.4% for women.^37^ Fat percentage was calculated as total body fat mass divided by body weight multiplied by 100. High fat percentage was classified for different age and sex groups: 40 to 59 years: more than 29% male and more than 41% female; 60 to 79 years: more than 31% male and more than 43% female.^38^

### Assessment of All-Cause Mortality

Information on vital status was obtained from general practitioners and the central registry of the Municipality of Rotterdam. Follow-up time started at the date of DXA scan, assessed between March 1, 2009, and June 1, 2014, and ended at the date of death or the end of the study (October 20, 2022), whichever came first.

### Assessment of Covariates

For each participant in this study, we retrieved information on the following covariates from questionnaires and interviews: age (categorized into <70 and ≥70 years), sex (male or female), and smoking status (categorized as current if they were smoking at the first visit, past if they had quit smoking, or never). Dietary intake was assessed with food-frequency questionnaries,^39^ from which we calculated protein intake standardized for energy intake and adjusted for body weight. Physical activity levels were assessed using a validated adapted version of the Longitudinal Aging Study Amsterdam Physical Activity Questionnaire and expressed in metabolic equivalent of task hours per week.^40^

Fasting blood samples were collected at the study center. Full blood cell counts were performed (Coulter Ac∙T diff2 Hematology Analyzer, Beckman Coulter). A homeostasis model assessment of insulin resistance score (HOMA-IR) was calculated using the following formula: fasting glucose × fasting insulin / 22.5.^41^ Triglycerides and glucose index was calculated based on the following formula: [ln(fasting triglycerides) × (fasting glucose) / 2].^42^ Estimated glomerular filtration rate was calculated with calibrated creatinine values using the equation from the Chronic Kidney Disease Epidemiology Collaboration.

The total number of comorbidities per individual was defined as 0, 1, 2 or more, or unknown (at least 1 missing). Eleven prevalent comorbidities were included (eTable 3 in Supplement 1).

### Statistical Analysis

Statistical analyses were performed from January 1 to April 1, 2023, using R, version 1.4.1106 (R Foundation for Statistical Computing) using the packages survival and VennDiagram. First, descriptive characteristics were summarized for all participants stratified by SO categories and sex. We calculated the prevalence of sarcopenia and SO and provided 95% CIs using the Wilson score method for a binomial proportion. All the analyses on sarcopenia by using the EWGSOP2 were included in the eAppendix in Supplement 1.

Second, survival probability was described using Kaplan-Meier curves for participants across prespecified sarcopenia and SO categories. For all-cause mortality as the primary outcome, associated with sarcopenia and SO, 2 Cox proportional hazards regression models were performed. Model 1 was sex and age adjusted, and model 2 was additionally adjusted for BMI. In model 2, we fitted an interaction term to investigate effect modification by BMI (sarcopenia × BMI and SO × BMI, respectively) on sarcopenia or SO. The results were expressed as hazard ratio (HRs) and their 95% CIs. We confirmed the assumptions of proportional hazards by statistical evaluation of Schoenfeld residual plots, inspecting for symmetry over time and *P* values. A 2-sided *P* < .05 was considered to indicate significance in all analyses.

We conducted a sensitivity analysis by including additional adjustments for comorbidities, smoking status, HOMA-IR, triglycerides and glucose index, physical activity, and protein intake because these variables have been previously associated with mortality risk and sarcopenia and SO.^43,44^ Obesity has been classically considered as a BMI of 30 or higher^45^; thus, we reran analyses with obesity based on a BMI of 30 or higher. Moreover, because age is a crucial driver of sarcopenia and SO, we stratified our analysis for age group (<70 vs ≥70 years). Additionally, we performed accelerated failure time models to complement the survival analysis as described in the eMethods in Supplement 1. Moreover, we presented graphically the overlapping categories of sarcopenia and SO by using a Venn diagram.

## Results

### Main Characteristics of the Study Population

**Figure 2.**
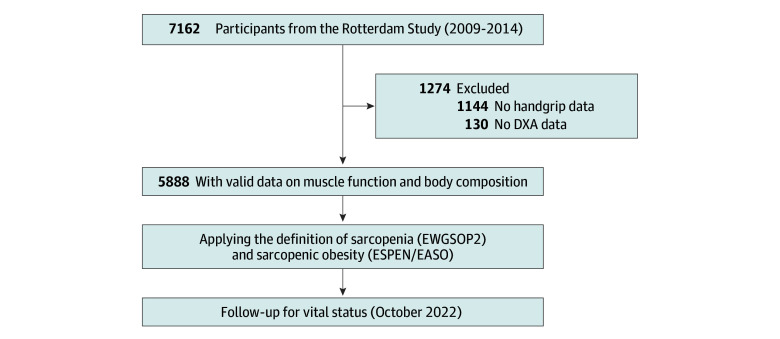
Flowchart of the Total Study Population DXA indicates dual-energy x-ray absorptiometry; ESPEN/EASO, European Society for Clinical Nutrition and Metabolism and the European Association for the Study of Obesity; EWGSOP2, European Working Group of Sarcopenia in Older People.

Between 2004 and 2019, a total of 7162 participants were recruited from the Rotterdam Study, and 5888 had full data available on SO (Figure 2). Baseline demographic and clinical characteristics of this study population are given in the Table. Moreover, sex-stratified characteristics are detailed in eTable 4 in Supplement 1. Biochemical characteristics are provided in eTable 5 in Supplement 1. In the total population, the mean (SD) age was 69.5 (9.1) years, mean (SD) BMI was 27.5 (4.3), 3343 (56.8%) were female, and 2545 (43.2%) male. Approximately 98% of the study population was of European ancestry, with the remaining 2% being of sub-Saharan African, East Asian, or multiethnic background. Given the largely homogeneous characteristics of the study population, ethnic background was not included as a factor in the analysis. During a median (IQR) of 9.9 (8.8-11.1) years of follow-up, 1538 deaths (26.1%) occurred.

**Table.** Main Characteristics of the Study Population^a^

Characteristic	Total population (N = 5888)	No sarcopenic-obesity (n = 3096)	Normal handgrip and altered BC (1 component) (n = 1903)	Normal handgrip and altered BC (2 components) (n = 109)	Low handgrip and normal BC (n = 441)	Low handgrip and altered BC (1 component) (n = 295)	Low handgrip and altered BC (2 components) (n = 44)
Age, mean (SD), y	69.5 (9.1)	68.4 (8.4)	67.7 (8.5)	71.6 (8.9)	78.6 (8.4)	76.9 (8.1)	77.2 (8.1)
Age ≥70 y	2690 (45.7)	1248 (40.3)	741 (38.9)	61 (56.0)	377 (85.5)	228 (77.3)	35 (79.5)
Sex							
Female	3343 (56.8)	2047 (66.1)	816 (42.9)	26 (23.9)	312 (70.7)	137 (46.4)	5 (11.4)
Male	2545 (43.2)	1049 (33.9)	1087 (57.1)	83 (76.1)	129 (29.3)	158 (53.6)	39 (88.6)
BMI, mean (SD)	27.5 (4.3)	25.6 (3.0)	30.3 (3.9)	34.2 (5.1)	25.2 (3.2)	29.7 (4.1)	30.4 (4.4)
Retired^b^	3546 (60.2)	1784 (57.6)	1071 (56.3)	85 (78.0)	334 (75.7)	232 (78.6)	40 (90.9)
Smoking status							
Past	3154 (53.6)	1545 (49.9)	1103 (58.0)	73 (67.0)	209 (47.4)	191 (64.7)	33 (75.0)
Current	713 (12.1)	419 (13.5)	212 (11.1)	14 (12.8)	53 (12.0)	11 (3.7)	4 (9.1)
Physical activity, median (IQR), MET h/wk	11.5 (5.0-22.0)	14.1 (6.0-24.7)	9.5 (4.1-19.0)	5.6 (3.0-12.0)	7.5 (3.5-17.5)	7.3 (3.0-16.0)	7.5 (3.9-14.7)
Daily protein intake, mean (SD), g/kg	1.1 (0.4)	1.2 (0.4)	0.9 (0.3)	0.8 (0.2)	1.2 (0.4)	0.9 (0.3)	0.8 (0.3)
HOMA-IR index, mean (SD)	3.7 (5.3)	2.8 (4.0)	4.8 (6.8)	6.4 (7.5)	3.0 (3.7)	4.4 (0.3)	5.6 (6.7)
Triglycerides and glucose ratio, mean (SD)	4.7 (0.3)	4.6 (0.2)	4.8 (0.3)	4.8 (0.3)	4.8 (0.2)	4.8 (0.2)	4.8 (0.2)
Comorbidities, No.							
1	983 (16.7)	669 (21.6)	231 (12.1)	5 (4.6)	52 (11.8)	22 (7.5)	4 (9.1)
≥2	4052 (68.8)	1945 (62.8)	1447 (76.0)	81 (74.3)	314 (71.2)	236 (80.0)	29 (65.9)
Sarcopenia status							
No	4865 (82.6)	2932 (94.7)	1841 (96.7)	92 (84.4)	NA	NA	NA
Only low ALM divided by height squared	243 (4.1)	164 (5.3)	62 (3.3)	17 (15.6)	NA	NA	NA
Probable sarcopenia	653 (11.1)	NA	NA	NA	363 (82.3)	261 (88.5)	29 (65.9)
Confirmed sarcopenia	127 (2.2)	NA	NA	NA	78 (17.7)	34 (11.4)	15 (34.1)

Abbreviations: ALM, appendicular lean mass; BC, body composition; BMI, body mass index (calculated as weight in kilograms divided by height in meters squared); HOMA-IR, Homeostatic Model Assessment for Insulin Resistance; MET, metabolic equivalent of task; NA, not applicable.

^a^ Data are presented as number (percentage) of participants unless otherwise indicated. Number (percentage) of missing values per variable are as follows: retired, 228 (3.9%); physical activity, 747 (12.7%); energy and protein intake, 1175 (20%); HOMA-IR, 120 (2.0%); and number of comorbidities, 563 (9.6%).

^b^ Retirement status was defined according to the following events: (1) receipt of an official retirement pension (statutory retirement due to age ≥65 years), (2) early retirement, and (3) renter or who is not retired but they are not working and living off the interest of their real assets (ie, properties). Original data without imputations are given.

### Prevalence of Sarcopenia Using the EWGSOP2 Definition

Within the total population, probable sarcopenia was found in 653 participants (11.1%; 95% CI, 10.3%-11.9%) and confirmed in 127 (2.2%; 95% CI, 1.8%-2.6%). Additionally, 243 participants (4.1%; 95% CI, 3.6%-4.6%) had normal muscle function with a low ALM divided by height squared.

### Prevalence of SO Using the ESPEN/EASO Definition

In the total population, 295 participants (5.0%; 95% CI, 4.4%-5.6%) had SO with 1 altered component of BC and 44 (0.8%; 95% CI, 0.6%-1.0%) had SO with 2 altered components of BC (Figure 1). In the subgroup of 2938 participants with BMIs of 27 or greater, 227 (7.7%; 95% CI, 6.7%-8.7%) had SO with 1 altered component of BC and 36 (1.2%; 95% CI, 0.8%-1.6%) had SO with 2 altered components of BC.

### Sarcopenia and All-Cause Mortality

In the total population, survival probability was lower in individuals with sarcopenia (both probable and confirmed) compared with those who were not classified as such (eFigure 2 in Supplement 1). We confirmed a significant interaction between sarcopenia and BMI on all-cause mortality. Consequently, we stratified the analyses by BMI (eFigure 2 in Supplement 1).

Adjusted for sex, age, and BMI, the HR for all-cause mortality was 1.29 (95% CI, 1.14-1.47) for individuals with probable sarcopenia and 1.93 (95% CI, 1.53-2.43) for those with confirmed sarcopenia (eTable 5 in Supplement 1). Participants with normal handgrip strength and low ALM divided by height squared were also at risk of death (HR, 1.66; 95% CI, 1.35-2.04). These associations remained similar for individuals with a BMI less than 27 (eTable 6 in Supplement 1).

### SO and All-Cause Mortality

**Figure 3.**
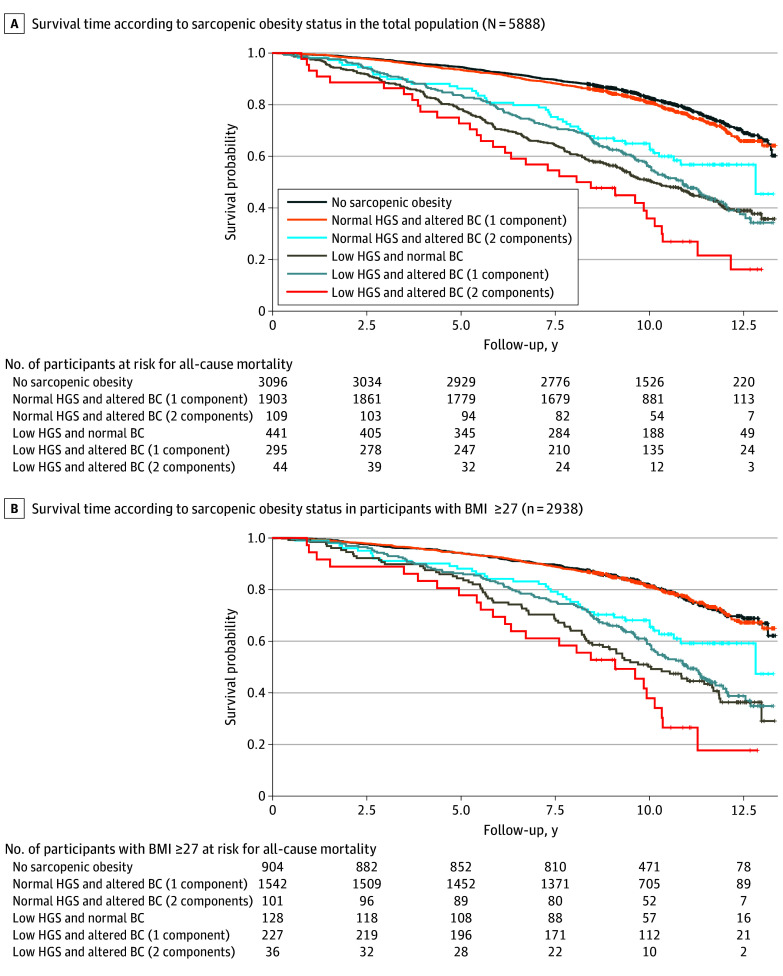
Kaplan-Meier Curves for Survival Time Kaplan-Meier curves for survival time are shown according to diagnostic criteria of sarcopenic obesity categories defined by the European Society for Clinical Nutrition and Metabolism and the European Association for the Study of Obesity consensus. BC indicates body composition; BMI, body mass index (calculated as weight in kilograms divided by height in meters squared); HGS, handgrip strength.

Among all participants, SO with 2 altered components of BC had worse survival compared with those without SO (Figure 3A). In participants with a BMI of 27 or greater, the descriptive Kaplan-Meier curves maintained the differences across SO categories (Figure 3B).

**Figure 4.**
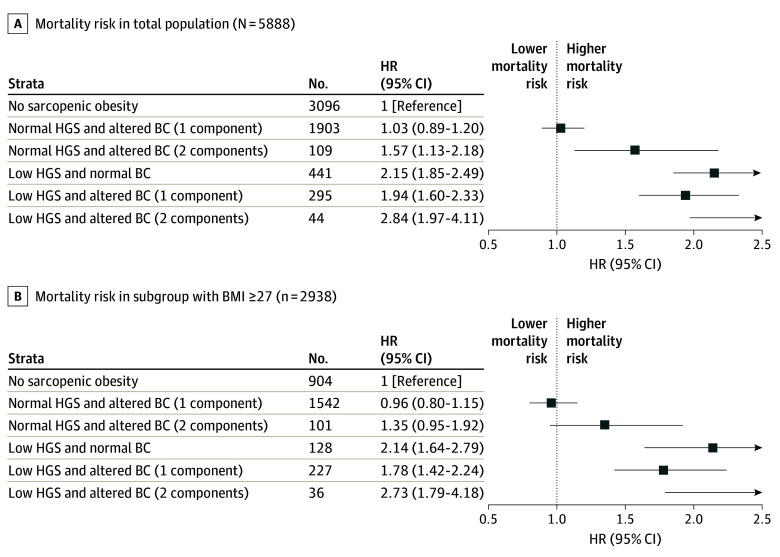
Association Between Sarcopenic Obesity and All-Cause Mortality Participants without sarcopenic obesity were used as the reference group. Data presented as adjusted hazard ratios (HRs) with 95% CIs (models adjusted accordingly for age, sex, and body mass index [BMI; calculated as weight in kilograms divided by height in meters squared]). BC indicates body composition; HGS, handgrip strength.

In the age- and sex-adjusted model, participants with SO and 2 altered components of BC had a higher risk of all-cause mortality (HR, 2.84; 95% CI, 1.97-4.11), as did SO participants with 1 altered component of BC (HR, 1.94; 95% CI, 1.60-2.33). Likewise, participants with low muscle function and normal BC had a significant association with mortality (HR, 2.15; 95% CI, 1.85-2.49). Participants with normal handgrip strength and altered BC (2 components) had 57% higher risk of all-cause mortality compared with those without SO (HR, 1.57; 95% CI, 1.13-2.18) (Figure 4A and B).

The proportional hazards assumptions for the association between SO and mortality were partially reached. Therefore, the validity of these results was established by using parametric accelerated failure time models (eMethods in Supplement 1).

### Sensitivity Analysis

Physical activity and protein intake attenuated the association between SO and all-cause mortality (eTable 6 in Supplement 1). When the analysis was repeated with a BMI cutoff of 30, the results were attenuated for SO with 1 altered component of BC (eTable 7 in Supplement 1). Results on accelerated failure time regression analysis were in line with the Cox proportional hazards regression models (eMethods in Supplement 1). eFigure 3 in Supplement 1 illustrates the overlap between different categories of sarcopenia and SO.

## Discussion

In this large, population-based cohort study, with the use of the contemporaneous consensus of sarcopenia and SO, more than 13% had low muscle function, and 6% had additionally 1 or 2 altered components of BC. Specifically, in participants with BMIs of 27 or greater, SO with 1 or 2 altered components of BC was even higher (7.7% and 1.2%, respectively). During 10 years of follow-up, participants with lower muscle function had a 29% increased risk of mortality from any cause compared with those without sarcopenia from the total population. Participants with low muscle function and a single altered component of BC had a 94% higher risk of all-cause mortality than those without SO. The effect estimates for participants with confirmed sarcopenia or SO with 2 affected components of BC were even more pronounced, although the prevalence was low (2.2% for confirmed sarcopenia and 1.0% for SO). All these associations were unaffected by age, sex, and BMI, highlighting the critical importance of future research for assessing the first component (ie, low muscle function) of both phenotypes (sarcopenia and SO) in clinical practice.

Previous studies have found a large variability in the prevalence of sarcopenia or SO depending on definitions, cutoffs, and population settings. For instance, Trajanoska et al^46^ reported a prevalence of sarcopenia of 4.4% in the Rotterdam Study while applying the initial consensus on sarcopenia (EWGSOP1), which prioritized muscle mass as the first defining component. Wagenaar et al^47^ described a relatively low prevalence of SO, ranging from 0.9% to 1.4%. They used 24-hour urine creatinine excretion to measure muscle mass in a Dutch population. In contrast, Vieira et al^16^ observed a relatively high prevalence of SO (ranging from 7.9% to 23.0%) in young patients who had undergone postbariatric surgery by using ESPEN/EASO criteria. Our study expands these prior findings by examining a larger population-based setting and adapting the combinations of low muscle function and altered BC components.

Our results are in line with previous studies linking sarcopenia and SO to an increase in all-cause mortality in older people.^2,27,28,30,37,48^ Zhang et al^27^ found that SO is a predictor of all-cause mortality in older people, particularly in those who were hospitalized. Similarly to the findings of Batsis et al,^49^ our study observed no association between those categories of normal muscle function accompanied by altered BC (low ALM-weight ratio and/or high fat percentage) and overall mortality. Nevertheless, compared with people without sarcopenia, those participants with low ASMI (adjusted by height squared) had an approximately 60% risk of death. Cruz-Jentoft et al^50^ recently explained how low lean mass is another condition that occurs at any age, regardless of sarcopenia, mainly associated with metabolic stress.

In this study, we used a BMI threshold of 27 or greater to define obesity, even though obesity has been officially defined by the World Health Organization as a standard BMI measure of 30 or greater.^45^ Accordingly, Donini et al^3^ advocated screening people with BMIs of 30 or greater to make an SO diagnosis. We have adapted 27 or greater as the BMI cutoff because previous studies have mentioned the impact on mortality and better correlation with body fat percentage.^35,51,52^ Our sensitivity analysis did not suggest any differences in the association of SO with all-cause mortality using a BMI cutoff of 30, except for SO with 1 altered component of BC. In addition, sarcopenia and obesity have complex interactions with multiple factors involved.^30,53^ When we additionally adjusted for physical activity and protein intake in our sensitivity analysis, their effect on mortality decreased but remained consistent among individuals with lower muscle function.

### Strengths and Limitations

This study has several strengths, including the large population-based setting with a long follow-up period, allowing a comprehensive examination of the research question, and the clinical assessment of sarcopenia and SO based on the most recent consensus (ie, EWGSOP2 and ESPEN/EASO). These consensus-based guidelines are particularly advantageous because they prioritize the identification of low muscle function as a primary criterion for screening and diagnosis, thereby enabling a clinical evaluation independent of BMI.

Nevertheless, potential limitations should be considered when interpreting the results. First, because there was a low prevalence of SO with 2 altered components of BC, stratification by sex was not possible. However, we applied sex-specific cutoffs for handgrip strength and ALM-weight ratio to define our exposed groups (sarcopenia and SO), and all our models were adjusted for sex. Second, we did not consider the specific cause of death analysis because the number of events in each category of SO was scarce. Third, most participants had European ancestry, limiting the generalizability of these results to other ethnicities.

## Conclusions

Sarcopenia and SO are common phenotypes in older people, and both conditions are associated with an increased risk of all-cause mortality. These results suggest that screening for SO might be implemented in primary care. In addition, early nonpharmacologic interventions, such as nutrition and exercise training, should be included to delay the onset of and to treat sarcopenia, especially SO. Likewise, comprehensive approaches encompassing nonpharmacologic and pharmacologic interventions may be more effective to treat both conditions. Finally, screening muscle function, as a first step of SO algorithm and as an independent comorbidity of obesity, might better assess the complexity of this metabolic disease.

## Notes

Supplement 1. **eFigure 1.** Correlation Between Fat % and Body Mass Index With Cut-Off Values **eTable 1.** Diagnostic Criteria of Sarcopenia and Cut-Off Values Based on the EWGSOP2 Consensus **eTable 2.** Diagnostic Criteria of Sarcopenic Obesity and Cut-Offs Based on the ESPEN/EASO Recommendations **eTable 3.** Eleven Main Prevalent Diseases Included in the Count of Comorbidities **eMethods.** Accelerated Failure Time Models **eTable 4.** Main Characteristics of the Study Population According to Sarcopenic Obesity Definition in Males and Females **eTable 5.** Biochemical and Characteristics of Study Population According to BMI and Sarcopenia Classification **eFigure 2.** Kaplan-Meier Curves for Survival Time According to Sarcopenia Status in A) All Population (N = 5888) and B) Population With BMI<27 kg/m^2^ **eTable 6.** Association Between Sarcopenia or Sarcopenic Obesity Categories and All-Cause Mortality **eTable 7.** Association Between SO and All-Cause Mortality in Participants With BMI ≥30 **eFigure 3.** Venn Diagram
eReferences

Supplement 2.
Data Sharing Statement

## References

[1] Cruz-Jentoft AJ, Sayer AA. Sarcopenia. Lancet. 2019;393(10191):2636-2646. doi:10.1016/S0140-6736(19)31138-9 31171417

[2] Beaudart C, Zaaria M, Pasleau F, Reginster JY, Bruyère O. Health outcomes of sarcopenia: a systematic review and meta-analysis. PLoS One. 2017;12(1):e0169548. doi:10.1371/journal.pone.0169548 28095426 PMC5240970

[3] Donini LM, Busetto L, Bischoff SC, . Definition and diagnostic criteria for sarcopenic obesity: ESPEN and EASO consensus statement. Obes Facts. 2022;15(3):321-335. doi:10.1159/000521241 35196654 PMC9210010

[4] Bahat G, Kilic C, Ozkok S, Ozturk S, Karan MA. Associations of sarcopenic obesity versus sarcopenia alone with functionality. Clin Nutr. 2021;40(5):2851-2859. doi:10.1016/j.clnu.2021.04.002 33940398

[5] Batsis JA, Haudenschild C, Crow RS, Gilliam M, Mackenzie TA. Sarcopenia definition outcome consortium—defined weakness and risk of falls: the National Health and Aging Trends Survey. Geriatr Gerontol Int. 2023;23(3):213-220. doi:10.1111/ggi.14548 36752357 PMC9992327

[6] Barazzoni R, Bischoff SC, Boirie Y, . Sarcopenic obesity: time to meet the challenge. Clin Nutr. 2018;37(6, pt A):1787-1793. doi:10.1016/j.clnu.2018.04.018 29857921

[7] Bischoff SC, Boirie Y, Cederholm T, . Towards a multidisciplinary approach to understand and manage obesity and related diseases. Clin Nutr. 2017;36(4):917-938. doi:10.1016/j.clnu.2016.11.007 27890486

[8] World Health Federation. World Obesity Atlas 2022. Updated December 13, 2022. Accessed February 8, 2024. https://www.worldobesity.org/resources/resource-library/world-obesity-atlas-2022

[9] Prado CM, Landi F, Chew STH, . Advances in muscle health and nutrition: a toolkit for healthcare professionals. Clin Nutr. 2022;41(10):2244-2263. doi:10.1016/j.clnu.2022.07.041 36081299

[10] Bahat G. Sarcopenic obesity: a hot yet under considered evolving concept. Eur Geriatr Med. 2022;13(5):1023-1024. doi:10.1007/s41999-022-00674-w 35831607

[11] Polyzos SA, Mantzoros CS. Sarcopenia: still in relative definition-penia and severe treatment-penia. Metabolism. 2024;150:155717. doi:10.1016/j.metabol.2023.155717 37923006

[12] Cruz-Jentoft AJ, Bahat G, Bauer J, ; Writing Group for the European Working Group on Sarcopenia in Older People 2 (EWGSOP2), and the Extended Group for EWGSOP2. Sarcopenia: revised European consensus on definition and diagnosis. Age Ageing. 2019;48(1):16-31. doi:10.1093/ageing/afy169 30312372 PMC6322506

[13] Cruz-Jentoft AJ, Montero-Errasquín B, Morley JE. Definitions of sarcopenia. Sarcopenia. John Wiley & Sons Ltd.; 2021:1-9. 10.1002/9781119597896

[14] Fernandes LV, Paiva AEG, Silva ACB, . Prevalence of sarcopenia according to EWGSOP1 and EWGSOP2 in older adults and their associations with unfavorable health outcomes: a systematic review. Aging Clin Exp Res. 2022;34(3):505-514. doi:10.1007/s40520-021-01951-7 34398438

[15] Gao Q, Mei F, Shang Y, . Global prevalence of sarcopenic obesity in older adults: a systematic review and meta-analysis. Clin Nutr. 2021;40(7):4633-4641. doi:10.1016/j.clnu.2021.06.009 34229269

[16] Vieira FT, Godziuk K, Lamarca F, . Sarcopenic obesity diagnosis by different criteria mid-to long-term post-bariatric surgery. Clin Nutr. 2022;41(9):1932-1941. doi:10.1016/j.clnu.2022.07.006 35947895

[17] Murawiak M, Krzymińska-Siemaszko R, Kaluźniak-Szymanowska A, . Sarcopenia, obesity, sarcopenic obesity and risk of poor nutritional status in Polish community-dwelling older people aged 60 years and over. Nutrients. 2022;14(14):2889. doi:10.3390/nu14142889 35889850 PMC9317847

[18] Gortan Cappellari G, Semolic A, Zanetti M, . Sarcopenic obesity in free-living older adults detected by the ESPEN-EASO consensus diagnostic algorithm: validation in an Italian cohort and predictive value of insulin resistance and altered plasma ghrelin profile. Metabolism. 2023;145:155595. doi:10.1016/j.metabol.2023.155595 37245728

[19] Scott D, Blyth F, Naganathan V, . Sarcopenia prevalence and functional outcomes in older men with obesity: comparing the use of the EWGSOP2 sarcopenia versus ESPEN-EASO sarcopenic obesity consensus definitions. Clin Nutr. 2023;42(9):1610-1618. doi:10.1016/j.clnu.2023.07.014 37481869

[20] Fumagalli C, Maurizi N, Day SM, ; SHARE Investigators. Association of obesity with adverse long-term outcomes in hypertrophic cardiomyopathy. JAMA Cardiol. 2020;5(1):65-72. doi:10.1001/jamacardio.2019.4268 31693057 PMC6865784

[21] Zhang X, Wang C, Dou Q, Zhang W, Yang Y, Xie X. Sarcopenia as a predictor of all-cause mortality among older nursing home residents: a systematic review and meta-analysis. BMJ Open. 2018;8(11):e021252. doi:10.1136/bmjopen-2017-021252 30420343 PMC6252774

[22] Bouchard DR, Dionne IJ, Brochu M. Sarcopenic/obesity and physical capacity in older men and women: data from the Nutrition as a Determinant of Successful Aging (NuAge)—the Quebec longitudinal Study. Obesity (Silver Spring). 2009;17(11):2082-2088. doi:10.1038/oby.2009.109 19373219

[23] Lee DC, Shook RP, Drenowatz C, Blair SN. Physical activity and sarcopenic obesity: definition, assessment, prevalence and mechanism. Future Sci OA. 2016;2(3):FSO127. doi:10.4155/fsoa-2016-0028 28031974 PMC5137918

[24] Atkins JL, Wannamathee SG. Sarcopenic obesity in ageing: cardiovascular outcomes and mortality. Br J Nutr. 2020;124(10):1102-1113. doi:10.1017/S0007114520002172 32616084

[25] Matsushita T, Nishioka S, Taguchi S, Yamanouchi A, Nakashima R, Wakabayashi H. Sarcopenic obesity and activities of daily living in stroke rehabilitation patients: a cross-sectional study. Healthcare (Basel). 2020;8(3):255. doi:10.3390/healthcare8030255 32781673 PMC7551564

[26] Yoshimura Y, Wakabayashi H, Nagano F, . The applicability of the ESPEN and EASO-defined diagnostic criteria for sarcopenic obesity in Japanese patients after stroke: prevalence and association with outcomes. Nutrients. 2022;14(19):4205. doi:10.3390/nu14194205 36235857 PMC9570818

[27] Zhang X, Xie X, Dou Q, . Association of sarcopenic obesity with the risk of all-cause mortality among adults over a broad range of different settings: a updated meta-analysis. BMC Geriatr. 2019;19(1):183. doi:10.1186/s12877-019-1195-y 31269909 PMC6610788

[28] Tian S, Xu Y. Association of sarcopenic obesity with the risk of all-cause mortality: a meta-analysis of prospective cohort studies. Geriatr Gerontol Int. 2016;16(2):155-166. doi:10.1111/ggi.12579 26271226

[29] Wannamethee SG, Atkins JL. Sarcopenic obesity and cardiometabolic health and mortality in older adults: a growing health concern in an ageing population. Curr Diab Rep. 2023;23(11):307-314. doi:10.1007/s11892-023-01522-2 37566368 PMC10640508

[30] Liu C, Wong PY, Chung YL, . Deciphering the “obesity paradox” in the elderly: a systematic review and meta-analysis of sarcopenic obesity. Obes Rev. 2023;24(2):e13534. doi:10.1111/obr.13534 36443946

[31] Donini LM, Busetto L, Bauer JM, . Critical appraisal of definitions and diagnostic criteria for sarcopenic obesity based on a systematic review. Clin Nutr. 2020;39(8):2368-2388. doi:10.1016/j.clnu.2019.11.024 31813698

[32] Gortan Cappellari G, Guillet C, Poggiogalle E, ; SOGLI Expert Panel. Sarcopenic obesity research perspectives outlined by the sarcopenic obesity global leadership initiative (SOGLI): proceedings from the SOGLI consortium meeting in Rome November 2022. Clin Nutr. 2023;42(5):687-699. doi:10.1016/j.clnu.2023.02.018 36947988

[33] Ikram MA, Brusselle G, Ghanbari M, . Objectives, design and main findings until 2020 from the Rotterdam Study. Eur J Epidemiol. 2020;35(5):483-517. doi:10.1007/s10654-020-00640-5 32367290 PMC7250962

[34] World Medical Association. World Medical Association Declaration of Helsinki: ethical principles for medical research involving human subjects. JAMA. 2013;310(20):2191-2194. doi:10.1001/jama.2013.281053 24141714

[35] Meeuwsen S, Horgan GW, Elia M. The relationship between BMI and percent body fat, measured by bioelectrical impedance, in a large adult sample is curvilinear and influenced by age and sex. Clin Nutr. 2010;29(5):560-566. doi:10.1016/j.clnu.2009.12.011 20359792

[36] Dodds RM, Syddall HE, Cooper R, . Grip strength across the life course: normative data from twelve British studies. PLoS One. 2014;9(12):e113637. doi:10.1371/journal.pone.0113637 25474696 PMC4256164

[37] Batsis JA, Barre LK, Mackenzie TA, Pratt SI, Lopez-Jimenez F, Bartels SJ. Variation in the prevalence of sarcopenia and sarcopenic obesity in older adults associated with different research definitions: dual-energy X-ray absorptiometry data from the National Health and Nutrition Examination Survey 1999-2004. J Am Geriatr Soc. 2013;61(6):974-980. doi:10.1111/jgs.12260 23647372

[38] Gallagher D, Heymsfield SB, Heo M, Jebb SA, Murgatroyd PR, Sakamoto Y. Healthy percentage body fat ranges: an approach for developing guidelines based on body mass index. Am J Clin Nutr. 2000;72(3):694-701. doi:10.1093/ajcn/72.3.694 10966886

[39] Voortman T, Kiefte-de Jong JC, Ikram MA, . Adherence to the 2015 Dutch dietary guidelines and risk of non-communicable diseases and mortality in the Rotterdam Study. Eur J Epidemiol. 2017;32(11):993-1005. doi:10.1007/s10654-017-0295-2 28825166 PMC5684301

[40] Koolhaas CM, Dhana K, Schoufour JD, . Physical activity and cause-specific mortality: the Rotterdam Study. Int J Epidemiol. 2018;47(5):1705-1713. doi:10.1093/ije/dyy058 29672692

[41] Salgado AL, Carvalho Ld, Oliveira AC, Santos VN, Vieira JG, Parise ER. Insulin resistance index (HOMA-IR) in the differentiation of patients with non-alcoholic fatty liver disease and healthy individuals. Arq Gastroenterol. 2010;47(2):165-169. doi:10.1590/S0004-28032010000200009 20721461

[42] Guerrero-Romero F, Simental-Mendía LE, González-Ortiz M, . The product of triglycerides and glucose, a simple measure of insulin sensitivity: comparison with the euglycemic-hyperinsulinemic clamp. J Clin Endocrinol Metab. 2010;95(7):3347-3351. doi:10.1210/jc.2010-0288 20484475

[43] Schoufour JD, Tieland M, Barazzoni R, . The relevance of diet, physical activity, exercise, and persuasive technology in the prevention and treatment of sarcopenic obesity in older adults. Front Nutr. 2021;8:661449. doi:10.3389/fnut.2021.661449 34109204 PMC8180560

[44] Eglseer D, Traxler M, Bauer S. Association between the intake of different protein sources and obesity coexisting with low handgrip strength in persons near retirement age. Nutrients. 2022;14(21):4684. doi:10.3390/nu14214684 36364946 PMC9653996

[45] World Health Organization. Obesity and overweight. 2021. Accessed February 10, 2024. https://www.who.int/news-room/fact-sheets/detail/obesity-and-overweight

[46] Trajanoska K, Schoufour JD, Darweesh SKL, . Sarcopenia and its clinical correlates in the general population: the Rotterdam Study. J Bone Miner Res. 2018;33(7):1209-1218. doi:10.1002/jbmr.3416 29502340

[47] Wagenaar CA, Dekker LH, Navis GJ. Prevalence of sarcopenic obesity and sarcopenic overweight in the general population: the Lifelines cohort study. Clin Nutr. 2021;40(6):4422-4429. doi:10.1016/j.clnu.2021.01.005 33485705

[48] Batsis JA, Mackenzie TA, Barre LK, Lopez-Jimenez F, Bartels SJ. Sarcopenia, sarcopenic obesity and mortality in older adults: results from the National Health and Nutrition Examination Survey III. Eur J Clin Nutr. 2014;68(9):1001-1007. doi:10.1038/ejcn.2014.117 24961545

[49] Batsis JA, Mackenzie TA, Emeny RT, Lopez-Jimenez F, Bartels SJ. Low lean mass with and without obesity, and mortality: results from the 1999-2004 National Health and Nutrition Examination Survey. J Gerontol A Biol Sci Med Sci. 2017;72(10):1445-1451. doi:10.1093/gerona/glx002 28207042 PMC5861857

[50] Cruz-Jentoft AJ, Gonzalez MC, Prado CM. Sarcopenia ≠ low muscle mass. Eur Geriatr Med. 2023;14(2):225-228. doi:10.1007/s41999-023-00760-7 36869279

[51] Heiat A, Vaccarino V, Krumholz HM. An evidence-based assessment of federal guidelines for overweight and obesity as they apply to elderly persons. Arch Intern Med. 2001;161(9):1194-1203. doi:10.1001/archinte.161.9.1194 11343442

[52] Visaria A, Setoguchi S. Body mass index and all-cause mortality in a 21st century U.S. population: a National Health Interview Survey analysis. PLoS One. 2023;18(7):e0287218. doi:10.1371/journal.pone.0287218 37405977 PMC10321632

[53] Ji T, Li Y, Ma L. Sarcopenic obesity: an emerging public health problem. Aging Dis. 2022;13(2):379-388. doi:10.14336/AD.2021.1006 35371597 PMC8947824

