# Supplementary material for: Sarcopenia and Sarcopenic Obesity and Mortality Among Older People
Source: JAMA Netw Open. 2024 Mar 25;7(3):e243604. doi: 10.1001/jamanetworkopen.2024.3604 (PMC10964118; doi:10.1001/jamanetworkopen.2024.3604)
Supplement: Supplement 1. — eFigure 1. Correlation Between Fat % and Body Mass Index With Cut-Off Values eTable 1. Diagnostic Criteria of Sarcopenia and Cut-Off Values Based on the EWGSOP2 Consensus eTable 2. Diagnostic Criteria of Sarcopenic Obesity and Cut-Offs Based on the ESPEN/EASO Recommendations eTable 3. Eleven Main Prevalent Diseases Included in the Count of Comorbidities eMethods. Accelerated Failure Time Models eTable 4. Main Characteristics of the Study Population According to Sarcopenic Obesity Definition in Males and Females eTable 5. Biochemical and Characteristics of Study Population According to BMI and Sarcopenia Classification eFigure 2. Kaplan-Meier Curves for Survival Time According to Sarcopenia Status in A) All Population (N = 5888) and B) Population With BMI<27 kg/m2 eTable 6. Association Between Sarcopenia or Sarcopenic Obesity Categories and All-Cause Mortality eTable 7. Association Between SO and All-Cause Mortality in Participants With BMI ≥30 eFigure 3. Venn Diagram eReferences [file jamanetwopen-e243604-s001.pdf]

Supplementary Online Content

Benz E, Pinel A, Guillet C, et al. Sarcopenia and sarcopenic obesity and mortality among older people. *JAMA Netw Open*. 2024;7(3):e243604.
doi:10.1001/jamanetworkopen.2024.3604

eFigure 1. Correlation Between Fat % and Body Mass Index With Cut-Off Values

eTable 1. Diagnostic Criteria of Sarcopenia and Cut-Off Values Based on the EWGSOP2 Consensus

eTable 2. Diagnostic Criteria of Sarcopenic Obesity and Cut-Offs Based on the ESPEN/EASO Recommendations

eTable 3. Eleven Main Prevalent Diseases Included in the Count of Comorbidities

eMethods. Accelerated Failure Time Models

eTable 4. Main Characteristics of the Study Population According to Sarcopenic Obesity Definition in Males and Females

eTable 5. Biochemical and Characteristics of Study Population According to BMI and Sarcopenia Classification

eFigure 2. Kaplan-Meier Curves for Survival Time According to Sarcopenia Status in A) All Population (N=5888) and B) Population With BMI <27 kg/m^2.

eTable 6. Association Between Sarcopenia or Sarcopenic Obesity Categories and All-Cause Mortality.

eTable 7. Association Between SO and All-Cause Mortality in Participants With BMI ≥30

eFigure 3. Venn Diagram

eReferences

This supplementary material has been provided by the authors to give readers additional information about their work.

© 2024 Benz E et al. *JAMA Network Open.*

efigure 1. Scatter plot with Pearson correlation (R) for males (red) and females (orange), showing the association of cut-off of BMI ≥27 kg/m^2 and the cut-off values of fat % according to sex and age (1).

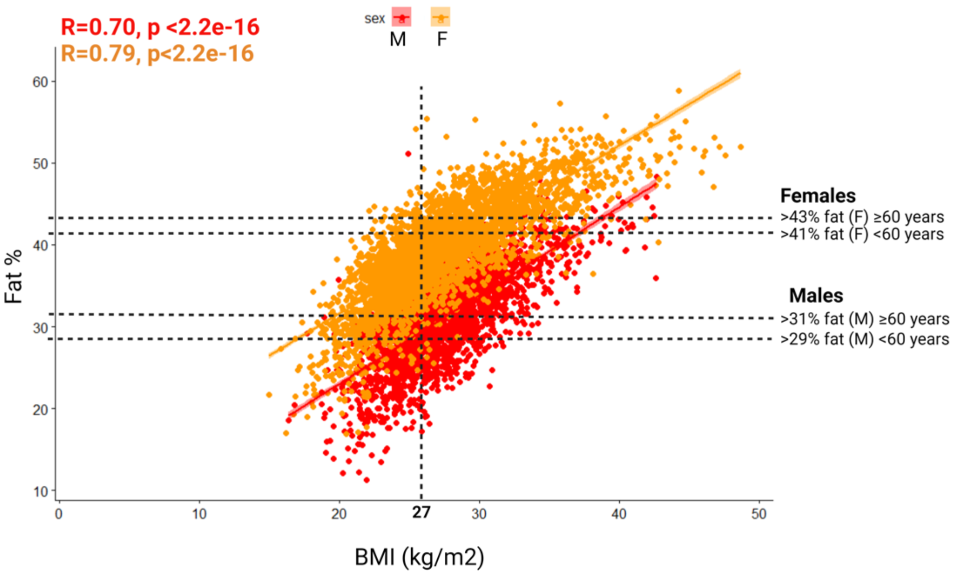

© 2024 Benz E et al. *JAMA Network Open.*

eTable 1. Diagnostic criteria of sarcopenia and cut-off values based on the EWGSOP2 consensus (2).

Definition	Outcome	Cut-off values	
		**Male**	**Female**
Probable sarcopenia	Low muscle strength: measured by hydraulic hand dynamometer	<27 kg	<16 kg
Sarcopenia	Low muscle strength **AND** Low lean mass: measured by DXA	<7.0 kg/m^2	<5.5 kg/m^2
Severe sarcopenia	Low muscle strength **AND** Low lean mass **AND** Low physical performance: measured by gait speed or Timed Up and Go Test (seconds)	≤0.8 m/s<br>≥20	≤0.8 m/s<br>≥20

Abbreviations: DXA: dual-energy X-ray absorptiometry; EWGSOP2: revised and updated European Working Group of Sarcopenia in Older People 2

© 2024 Benz E et al. *JAMA Network Open.*

eTable 2. Diagnostic criteria of sarcopenic obesity and cut-offs based on the ESPEN/EASO recommendations (3).

Stages	Outcome	Cut-off values	
		**Male**	**Female**
**1.Stage 1** Muscle function	Low muscle strength: measured by hydraulic hand dynamometer	<27 kg	<16 kg
**2. Stage 2** Body composition	Low muscle strength **AND**		
	Low lean mass adjusted for weight measured by DXA.	<25.7%	<19.4%
	High body fat percentage measured by DXA.	Age: 40-59: >29%<br>Age: 60-79: >31%	Age: 40-59: >41%<br>Age: 60-79: >43%

Abbreviations: DXA: dual-energy X-ray absorptiometry. ESPEN/EASO: European Society for Clinical Nutrition and Metabolism/European Association for the Study of Obesity.

© 2024 Benz E et al. *JAMA Network Open.*

eTable 3. Eleven main prevalent diseases included in the count of comorbidities.

	Total population	BMI ≥ 27 kg/m^2					
		**Sarcopenic obesity based on ESPEN/EASO**					
		No SO	Normal handgrip and altered BC (1 c)	Normal handgrip and altered BC (2 c)	Low handgrip and normal BC	Low handgrip and altered BC (1 c)	Low handgrip and altered BC (2 c)
	**n=5888**	n=904	n=1542	n=101	n=128	n=227	n=36
Comorbidities, n (%)							
T2D	873 (14.8)	164 (18.1)	296 (19.2)	30 (29.7)	36 (28.1)	52 (22.9)	17 (47.2)
Hypertension	4304 (73.1)	705 (78.0)	1247 (80.9)	95 (94.1)	120 (93.8)	214 (94.3)	33 (91.7)
CHD	524 (8.9)	60 (8.6)	158 (10.2)	15 (14.9)	14 (10.9)	35 (15.4)	9 (25.0)
Cancer	554 (9.4)	78 (8.6)	142 (9.2)	11 (10.9)	14 (10.9)	30 (13.2)	3 (8.3)
COPD	808 (13.7)	85 (9.4)	164 (10.6)	19 (18.8)	17 (13.3)	35 (15.4)	4 (11.1)
Asthma	441 (7.5)	59 (6.5)	137 (8.9)	14 (13.9)	9 (7.0)	27 (11.9)	3 (8.3)
Osteoporosis	3558 (60.4)	474 (52.4)	771 (50.0)	46 (45.5)	92 (71.9)	145 (63.9)	18 (50.0)
Steatosis	2051 (34.8)	388 (42.9)	888 (57.6)	64 (63.4)	49 (30.5)	126 (55.5)	18 (50.0)
NAFLD	1543 (26.2)	301 (33.3)	664 (43.1)	45 (44.6)	39 (30.5)	100 (44.1)	15 (41.7)
MAFLD	1974 (33.5)	388 (42.9)	887 (57.5)	64 (63.4)	49 (38.3)	126 (55.5)	18 (50.0)
Depression	517 (8.8)	73 (8.1)	137(8.9)	12 (11.9)	18 (14.1)	38 (16.7)	3 (8.3)

Definitions: 1) type 2 diabetes [T2D] was defined as fasting plasma glucose level ≥7 mmol/L, or a non-fasting plasma glucose level≥11.1 mmol/L or the use of blood glucose-lowering medication; 2) hypertension was defined as systolic blood pressure ≥140 mmHg or diastolic blood pressure ≥90 mmHg or use of antihypertensive medication; 3) coronary heart disease was defined as myocardial infarction, coronary artery bypass grafting or percutaneous coronary intervention; 4) cancer [clinical-based]; 5) chronic obstructive pulmonary disease (COPD) [clinical and spirometry based]; 6) asthma [clinical-based]; 7) Osteoporosis: osteopenia and osteoporosis were defined according to World Health Organization criteria (WHO) as a t-score between -1 to -2.5, and below or equal to -2.5, respectively; 8) steatosis [clinical-based]; 9) non-alcoholic fatty liver disease (NAFLD) [clinical-based]; 10) metabolic associated liver disease (MAFLD) [clinical-based]; 11) Depression: depression symptoms were validated Center for Epidemiologic Studies Depression Scale (CES-D) and scores ≥16 were considered as major depressive symptoms.

© 2024 Benz E et al. *JAMA Network Open.*

eMethods. Accelerated failure time models

Based on the multivariable Cox model (model 2) we fitted an accelerated failure time (AFT) regression model to analyse the association between the expected time of death and sarcopenia or sarcopenic obesity categories compared to participants without these conditions.

Our strategy to build this model was as follows. First, we checked the distribution of the error terms for our outcomes separately (sarcopenic-obesity and sarcopenia). It means that we selected the distribution that fits our data and by which the residuals are normally distributed. We checked the error terms of model 2 with different distributions (i.e., gaussian, lognormal, exponential). Because these error terms are influenced by censoring, we used the Kaplan-Meier estimator to create a survival function for the residual. Then, we plotted the distribution of the error terms and the final distribution (exponential) chosen for our outcomes (**eMethods Figure 1**). We used likelihood ratio tests to check overall statistic significance, compare the models with and without interactions and the linearity of the association.

Results of accelerated failure time models (eMethods tables 1 and 2).

Compared to subjects without sarcopenia, participants with sarcopenia (probable and confirmed) (β=-1.05 [95%CI: -1.55; -0.55 and β=-2.73 [95%CI: -3.61; -1.85]) and low ALM/height^2 (β=-1.90 [-2.70; -1.11]) had an accelerated time of death independent of age, sex and BMI (model 2)
In total population, those participants with low HGS and 2 altered BC components (β=-2.27 [95% CI: -3.90; -1.05]) or 1 altered BC component (β=-0.91[95%CI: -1.65; -0.18]) had an accelerated failure time to death than those without SO, independent of sex, age and BMI.

© 2024 Benz E et al. *JAMA Network Open.*

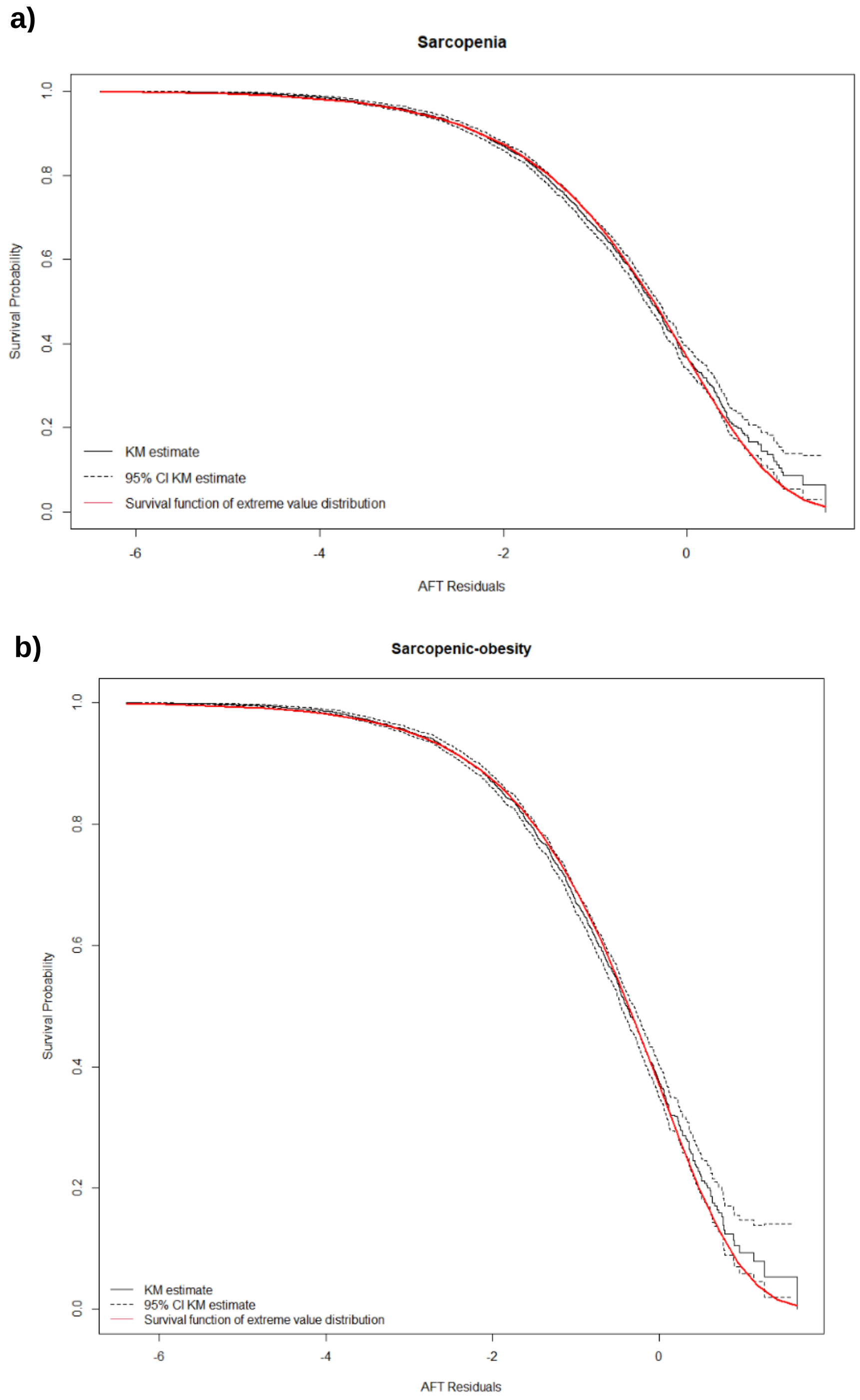

eMethods figure 1. Kaplan-Meier estimator of the residual with extreme distribution in a) sarcopenia and b) SO. The red and black lines are almost the same. It means that the extreme distribution can be a good distribution for these outcomes.

© 2024 Benz E et al. *JAMA Network Open.*

eMethods table 1). Association between sarcopenia and all-cause mortality.

	No sarcopenia n=4865	Low ALM divided by height ² n=243	Probable sarcopenia n=653	Confirmed sarcopenia n=127
β		β [95% CI]	β [95%CI]	β [95%CI]
Model 1	Ref.	-1.58 [-2.34; -0.82]	-1.08 [-1.58; -0.58]	-2.42 [-3.27; -1.57]
Model 2	Ref.	-1.90 [-2.70; -1.11]	-1.05 [-1.55; -0.55]	-2.73 [-3.61; -1.85]

Data presented the coefficients (β) as log failure time with 95% CI. Participants without sarcopenia were used as reference. Model 1: adjusted for sex and age; model 2: additionally adjusted for BMI.

© 2024 Benz E et al. *JAMA Network Open.*

eMethods table 2). Association between sarcopenia and all-cause mortality.

	No SO	Normal handgrip and altered BC (1c)<br><br>n=1903	Normal handgrip and altered BC (2c)<br><br>n=109	Low handgrip and Normal BC<br><br>n=441	Low handgrip and altered BC (1c)<br><br>n=295	Low handgrip and altered BC (2 c)<br><br>n=44
β		β [95% CI]	β [95%CI]	β [95%CI]	β [95%CI]	β [95%CI]
Model 1	Ref.	-0.24<br>[-0.72; 0.24]	-1.04<br>[-2.22; 0.14]	-1.35<br>[1.94; -0.76]	-0.97<br>[-1.65; -0.28]	-2.54<br>[-3.92; -1.15]
Model 2	Ref.	-0.19<br>[-0.74; 0.37]	-0.95<br>[-2.21; 0.31]	-1.36<br>[-1.94; -0.77]	-0.91<br>[-1.65; -0.18]	-2.47<br>[-3.90; -1.05]

Data presented the coefficients (β) as log failure time with 95% CI. Participants without SO were used as reference. Model 1: adjusted for sex and age; model 2: additionally adjusted for BMI.

© 2024 Benz E et al. *JAMA Network Open.*

eTable4. a) Main characteristics of the study population according to Sarcopenic obesity definition in males

	Total male population n=2545	No sarcopenic-obesity n=1049	Normal handgrip and altered BC (1 component) n=1087	Normal handgrip and altered BC (2 components) n=83	Low handgrip and normal BC n=129	Low handgrip and altered BC (1 component) n=158	Low handgrip and altered BC (2 components) n=39
Age, years<br>≥70 years, n (%)	69.3±8.9<br>1142 (44.9)	68.4±8.3<br>426 (40.6)	67.6±8.2<br>410 (37.7)	73.4±8.8<br>55 (66.3)	76.9±9.5<br>97 (75.2)	77.1±8.4<br>122 (77.2)	78.2±7.4<br>32 (82.1)
BMI, kg/m^2	27.4±3.6	25.5±2.5	29.1±3.2	33.0±4.5	24.5±2.7	27.9±2.7	30.1±4.4
Retired, n (%)	1694 (66.6)	665 (63.5)	673 (61.9)	71 (85.5)	110 (85.3)	136 (86.1)	38 (97.4)
Smoking status, n (%)<br>*Past*<br>*Current*	 1595 (62.7)<br>306 (12.0)	 617 (58.8)<br>136 (13.0)	 691 (63.6)<br>129 (11.9)	 63 (75.9)<br>8 (9.6)	 78 (60.5)<br>21 (16.3)	 115 (72.8)<br>8 (5.1)	 31 (79.5)<br>4 (10.3)
PA, MET hours/week	10.3 [4.8; 19.2]	12.2 [5.8; 21.5]	9.4 [4.3; 17.8]	5.0 [2.6; 12.0]	9.3 [4.0; 17.6]	7.7 [3.6; 16.9]	7.0 [4.2; 13.6]
Protein intake, g/k/d	1.1±0.3	1.1±0.4	1.0±0.3	0.8±0.3	1.2±0.4	0.9±0.3	0.8±0.3
HOMA-IR index	3.9±4.8	2.8±3.6	4.8±5.3	6.6±8.0	2.6±2.4	4.3±5.3	5.9±7.1
TyG	4.7±0.3	4.6±0.3	4.8±0.3	4.8±0.3	4.6±0.2	4.7±0.2	4.8±0.2
Comorbidities, n (%)<br>*1*<br>*≥2*	 389 (15.3)<br>1787 (70.2)	 210 (20.0)<br>673 (64.2)	 142 (13.1)<br>812 (74.7)	 2 (2.4)<br>62 (74.7)	 19 (14.7)<br>90 (69.8)	 13 (8.2)<br>124 (78.5)	 3 (7.7)<br>26 (66.7)
Sarcopenia status<br>*No*<br>*Only low ALM/height^2*<br>*Probable sarcopenia*<br>*Confirmed sarcopenia*	 2076 (81.6)<br>143 (5.6)<br>248 (9.7)<br>78 (3.1)	 973 (92.8)<br>76 (7.2)<br>-<br>-	 1036 (95.3)<br>51 (4.7)<br>-<br>-	 67 (80.7)<br>16 (19.3)<br>-<br>-	 -<br>-<br>93 (72.1)<br>36 (27.9)	 -<br>-<br>130 (82.3)<br>28 (17.7)	 -<br>-<br>25 (64.1)<br>14 (35.9)

© 2024 Benz E et al. *JAMA Network Open.*

eTable4. b) Main characteristics of the study population according to Sarcopenic obesity definition in females

	Total female population<br>n=3343	No sarcopenic-obesity<br>n=2047	Normal handgrip and altered BC (1 component)<br>n=816	Normal handgrip and altered BC (2 components)<br>n=26	Low handgrip and normal BC<br>n=312	Low handgrip and altered BC (1 component)<br>n=137	Low handgrip and altered BC (2 components)<br>n=5
Age, years<br>≥70 years, n (%)	69.6±9.2<br>1548 (46.3)	68.4±8.4<br>822 (40.2)	67.8±8.8<br>331 (40.6)	65.9±6.3<br>6 (23.1)	79.3±7.8<br>280 (89.7)	76.8±8.3<br>106 (77.4)	69.0±8.8<br>3 (60.0)
BMI, kg/m^2	27.5±4.7	25.6±3.3	32.0±4.2	37.8±5.0	25.5±3.4	31.9±4.3	32.7±3.1
Retired, n (%)	1852 (55.4)	1118 (54.6)	398 (48.8)	14 (53.8)	224 (71.8)	96 (70.1)	2 (40.0)
Smoking status, n (%)<br>*Past*<br>*Current*	 1559 (56.6)<br>407 (12.2)	 928 (45.3)<br>283 (13.8)	 412 (50.5)<br>83 (10.2)	 10 (38.5)<br>6 (23.1)	 131 (42.0)<br>32 (10.3)	 76 (55.5)<br>3 (2.2)	 3 (60.0)<br>2 (40.0)
PA, MET hours/week	12.5 [5.0; 24.3]	15.5 [6.3; 26.3]	9.5 [4.0; 21.3]	8.4 [3.8; 18.9]	7.0 [3.1; 17.5]	6.8 [3.0; 13.3]	19.9 [10.6; 20.6]
Protein intake, g/k/d	1.1±0.4	1.2±0.4	0.9±0.3	0.9±0.2	1.2±0.5	0.9±0.3	0.6±0.1
HOMA-IR index	3.5±5.6	2.9±4.2	4.8±8.4	5.7±5.8	3.1±4.1	4.6±4.2	3.5±1.3
TyG	4.7±0.2	4.6±0.2	4.8±0.2	4.8±0.2	4.7±0.3	4.8±0.2	4.8±0.2
Comorbidities, n (%)<br>*1*<br>≥2	 594 (17.8)<br>2265 (67.8)	 459 (22.4)<br>1272 (62.1)	 89 (10.9)<br>635 (77.8)	 3 (11.5)<br>19 (73.1)	 33 (10.6)<br>224 (71.8)	 9 (6.6)<br>112 (81.8)	 1 (20.0)<br>3 (60.0)
Sarcopenia status<br>*No*<br>*Only low ALM/height^2*<br>*Probable sarcopenia*<br>*Confirmed sarcopenia*	 2789 (83.4)<br>100 (3.0)<br>405 (12.1)<br>49 (1.5)	 1959 (95.7)<br>88 (4.3)<br>-<br>-	 805 (98.7)<br>11 (1.3)<br>-<br>-	 25 (96.2)<br>1 (3.8)<br>-<br>-	 -<br>-<br>270 (86.5)<br>42 (13.5)	 -<br>-<br>131 (95.6)<br>6 (4.4)	 -<br>-<br>4 (80.0)<br>1 (20.0)

© 2024 Benz E et al. *JAMA Network Open.*

eTable 5. Biochemical and characteristics of study population (n=5888).

	Total population	b) BMI ≥27kg/m² (n=2938)						
		**Sarcopenic obesity based on ESPEN/EASO**						
	n=5888	BMI≥27 kg/m² n=2938	No SO n=904	Normal handgrip and altered BC (1 c) n=1542	Normal handgrip and altered BC (2 c) n=101	Low handgrip and normal BC n=128	Low handgrip and altered BC (1 c) n=227	Low handgrip and altered BC (2 c) n=36
Cholesterol, mmol/L	5.5±1.1	5.4±1.1	5.4±1.1	5.4 ±1.1	5.1±1.1	5.4±1.2	5.2±1.1	4.9±1.1
HDL-chol, mmol/L	1.5±0.4	1.4±0.4	1.4±0.4	1.4±.0.4	1.3±0.3	1.4±0.4	1.4±0.3	1.3±0.3
Triglycerides, mmol/L	1.4±0.8	1.6±0.8	1.5±0.8	1.7±0.9	1.6±0.7	1.7±1.1	1.5±0.7	1.7±0.7
Glucose, mmol/L	5.8±1.0	6.1±1.4	5.9±1.3	6.1±1.4	6.5±1.7	5.9±1.3	6.2±1.5	7.0±2.5
Insulin, mmol/L	73.0 [51.0; 106.0]	92.0 [66.0; 131.0]	80.0 [59.0; 113.3]	100 [71.0; 142.0]	116 [86.0; 162.0]	81.0 [61.0; 114.5]	92.0 [67.0; 131.3]	105.0 [68.5; 128.5]
TyG index	4.7 ±0.3	4.8±0.3	4.7±0.3	4.8±0.2	4.8±0.3	4.8±0.3	4.8±0.2	4.9±0.2
ASAT, U/L	24.0 [21.0; 28.0]	24.0 [21.0; 28.5]	24.0 [21.0; 28.0]	24.0 [21.0; 29.0]	24.0 [20.0; 29.0]	24.0 [21.5; 28.0]	25.0 [21.0; 28.0]	24.0 [21.0; 28.3]
ALAT, U/L	19.0 [15.0; 24.0]	20.0 [15.0; 26.0]	19.0 [15.0; 25.0]	21.0 [16.0; 27.0]	20.0 [15.0; 29.0]	18.0 [14.5; 22.0]	18.0 [15.0; 24.0]	19.0 [14.0; 27.0]
Bilirubin, µmol/L	8.0 [6.0; 11.0]	8.0 [6.0; 10.5]	8.0 [6.0; 11.0]	8.0 [6.0; 11.0]	8.0 [6.0; 11.0]	8.0 [6.0; 10.0]	8.0 [6.0; 10.0]	9.0 [7.0; 12.0]
Creatinine, µmol/L	77.0 [67.0;90.0]	78.0 [68.0;91.0]	76.0 [66.0; 87.0]	80.0 [69.0; 92.0]	82.0 [73.0; 95.0]	76.0 [65.0; 90.0]	82.0 [70.0; 99.0]	79.5 [67.8; 95.8]
SII index	461 [338; 631]	462 [347; 628]	446 [337;602]	458 [345; 615]	513 [395; 644]	543 [396; 725]	520 [361; 717]	443 [357; 597]
e GFR, mL/min/1.73m²	75.4±15.0	74.7±15.3	74.7±14.0	76.2±15.0	76.0±17.4	67.7±16.0	67.4±17.6	75.8±17.1

Data are presented as means ± standard deviation (SD) or median values with corresponding interquartile ranges [IQR]. *Abbreviations*: ALM: appendicular lean mass; c: components; BC: body composition; HDL: high-density lipoprotein; ASAT: Aspartate transaminase; ALAT: Alanine aminotransferase; SII: systemic immune-inflammation index; e GFR: estimated glomerular filtration rate. *Missing values:* SII: 99 (1.7%); cholesterol, triglycerides, creatinine and glucose: 112 (1.9%); bilirubin and eGFR: 113 (1.9%); insulin, ASAT and ALAT:114 (1.9%). SII was calculated from the platelets (P ×109/l), granulocytes, as a proxy for neutrophils (N×109//) and lymphocytes (L×109/l), by using the formula: SII=P×N/L (4). Serum cholesterol, triglycerides, glucose, insulin, serum creatinine, alanine aminotransferase (ALAT), aspartate transaminase (ASAT), hemoglobin, and total bilirubin were measured using automatic enzyme procedures. Triglycerides and glucose index (TyG) was calculated based on this formula: TyG=[ln(fasting triglycerides) (mg/dl) × (fasting glucose) (mg/dl)/2] (5).

© 2024 Benz E et al. *JAMA Network Open.*

eFigure 2. Kaplan-Meier curves for survival time according to sarcopenia categories defined by EWGSOP2 consensus in **a)** all population (n=5888) and **b)** participants with BMI <27 kg/m^2 (n=2950)

a)

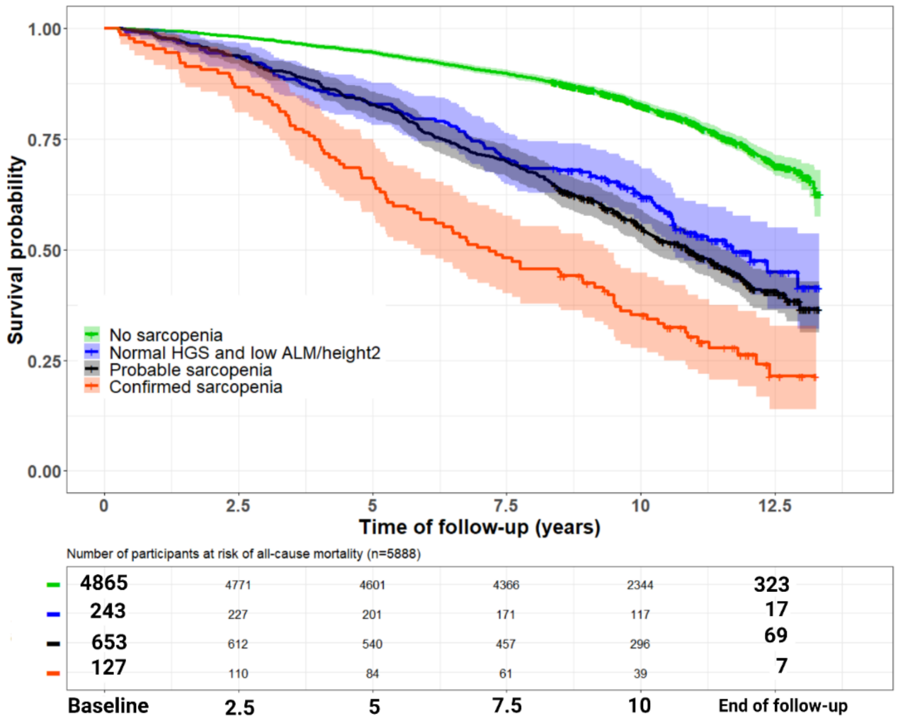

b)

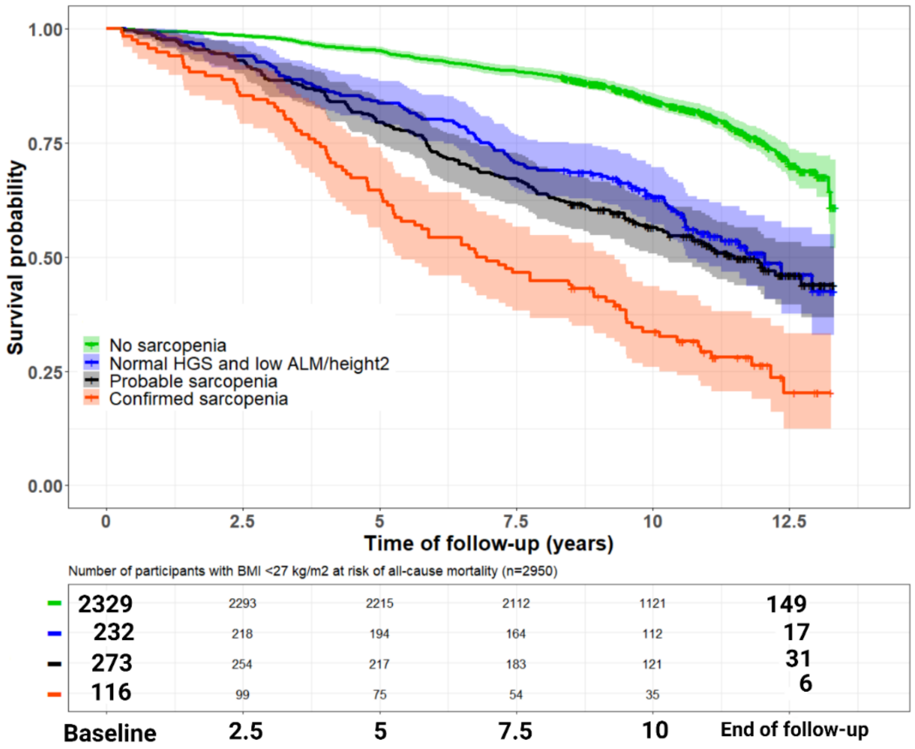

© 2024 Benz E et al. *JAMA Network Open.*

eTable 6. Association between sarcopenia or sarcopenic obesity categories and all-cause mortality in all population.

	Definition of sarcopenia based on EWGSOP2 (n=5888)				Definition of sarcopenic obesity based on ESPEN/EASO (n=5888)					
	No sarco-penia	Low ALM divided by height squared	Probable sarcopenia	Confirmed sarcopenia	No SO	Normal handgrip and altered BC (1 c)	Normal handgrip and altered BC (2 c)	Low handgrip and normal BC	Low handgrip and altered BC (1 c)	Low handgrip and altered BC (2 c)
	n=4865	n=243	n=653	n=127	n=3096	n=1903	n=109	n=441	n=295	n=44
		HR [95% CI]	HR [95% CI]	HR [95% CI]		HR [95% CI]	HR [95% CI]	HR [95% CI]	HR [95%CI]	HR [95% CI]
Model 2	Ref.	**1.66 [1.35; 2.04]**	**1.29 [1.13; 1.47]**	**1.93 [1.53; 2.43]**	Ref.	1.03 [0.89;1.20]	**1.57 [1.13; 2.18]**	**2.15 [1.85; 2.49]**	**1.94 [1.60; 2.33]**	**2.84 [1.97; 4.10]**
+comorbidities		**1.66 [1.35; 2.05]**	**1.29 [1.13; 1.47]**	**1.93 [1.53; 2.43]**		1.00 [0.86; 1.15]	**1.47 [1.05; 2.04]**	**2.06 [1.77; 2.39]**	**1.87 [1.55; 2.26]**	**2.72 [1.88; 3.94]**
+smoking status		**1.60 [1.30; 1.97]**	**1.30 [1.14 1.48]**	**1.86 [1.47; 2.34]**		1.03 [0.89; 1.19]	**1.53 [1.10; 2.14]**	**2.15 [1.85; 2.49]**	**1.98 [1.64; 2.39]**	**2.81 [1.95; 4.07]**
+ HOMA-IR		**1.62 [1.31; 2.01]**	**1.29 [1.13; 1.47]**	**1.88 [1.48; 2.39]**		1.00 [0.86; 1.16]	**1.46 [1.03; 2.05]**	**2.09 [1.80; 2.44]**	**1.90 [1.57; 2.30]**	**2.77 [1.91; 4.03]**
+TyG		**1.61 [1.30; 1.99]**	**1.28 [1.12; 1.46]**	**1.88 [1.48; 2.39]**		0.99 [0.86; 1.15]	**1.46 [1.04; 2.06]**	**2.08 [1.79; 2.42]**	**1.88 [1.56; 2.28]**	**2.69 [1.85; 3.92]**
+ PA		**1.57 [1.24; 1.98]**	**1.24 [1.06; 1.44]**	**1.95 [1.48; 2.56]**		0.99 [0.85; 1.17]	1.26 [0.85; 1.87]	**1.96 [1.65; 2.33]**	**1.74 [1.39; 2.18]**	**2.29 [1.43; 3.67]**
+Protein intake		**1.53 [1.21; 1.93]**	**1.22 [1.05; 1.41]**	**1.90 [1.43; 2.53]**		0.99 [0.84; 1.16]	1.41 [0.97; 2.03]	**2.03 [1.70; 2.41]**	**1.71 [1.37; 2.13]**	**3.05 [2.00; 4.66]**
*Stratification by age**										
<70 years (n=3198)	n=2966<br>Ref	n=92<br>**2.50 [1.52; 4.09]**	n=119<br>1.54 [0.90; 2.65]	n=21<br>**8.14 [4.43; 14.97]**	n=1848<br>Ref.	n=1162<br>**1.38 [1.06; 1.80]**	n=48<br>**2.60 [1.32; 5.15]**	n=64<br>**2.79 [1.50; 5.18]**	n=67<br>**2.36 [1.27; 4.39]**	n=9<br>**5.17 [1.64; 16.34]**
≥70 years (n=2690)	n=1899<br>Ref.	n=151<br>**1.73 [1.39; 2.15]**	n=534<br>**1.99 [1.75; 2.27]**	n=106<br>**2.85 [2.26; 3.59]**	n=1248<br>Ref.	n=741<br>0.92 [0.80; 1.07]	n=61<br>1.37 [0.97; 1.93]	n=377<br>**2.05 [1.75; 2.39]**	n=228<br>**1.82 [1.52; 2.19]**	n=35<br>**2.61 [1.79; 3.81]**
Stratification by BMI#					-	-	-	-	-	-
<27 kg/m^2 (n=2950)	n=2329<br>Ref.	n=232<br>**1.63 [1.31; 2.04]**	n=273<br>**1.38 [1.13; 1.70]**	n=116<br>**1.89 [1.47; 2.44]**						
≥27 kg/m^2 (n=2938)					n=904<br>Ref.	n=1542<br>0.96 [0.80; 1.15]	n=101<br>1.35 [0.95; 1.92]	n=128<br>**2.14 [1.64; 2.79]**	n=227<br>**1.78 [1.42; 2.24]**	n=36<br>**2.73 [1.79; 4.18]**

Model 2 adjusted for sex, age and BMI. *Models adjusted only for sex. #Models adjusted for sex and age. Significant estimates (p<0.05) are in bold.

© 2024 Benz E et al. *JAMA Network Open.*

eTable 7. Association between SO and all-cause mortality in participants with BMI ≥30 (n=1391).

	**Unadjusted**	**Model 1**
Normal handgrip and altered BC (1 component) (n=904)	0.87 [0.64; 1.18]	0.82 [0.60; 1.11]
Normal handgrip and altered BC (2 components) (n=83)	1.38 [0.87; 2.19]	1.03 [0.63; 1.67]
Low handgrip and normal BC (n=35)	2.82 [1.72; 4.62]	**1.94 [1.17; 3.19]**
Low handgrip and altered BC (1 component) (n=125)	1.81 [1.25; 2.64]	1.40 [0.96; 2.04]
Low handgrip and altered BC (2 components) (n=21)	3.27 [1.75; 6.02]	**2.10 [1.11; 3.99]**

Model 1: adjusted for sex and age

© 2024 Benz E et al. *JAMA Network Open.*

eFigure 3. Venn diagram of participants classified according to the EWGSOP2 as probable or confirmed sarcopenia and to ESPEN/AEASO as low muscle strength with and without altered body composition.

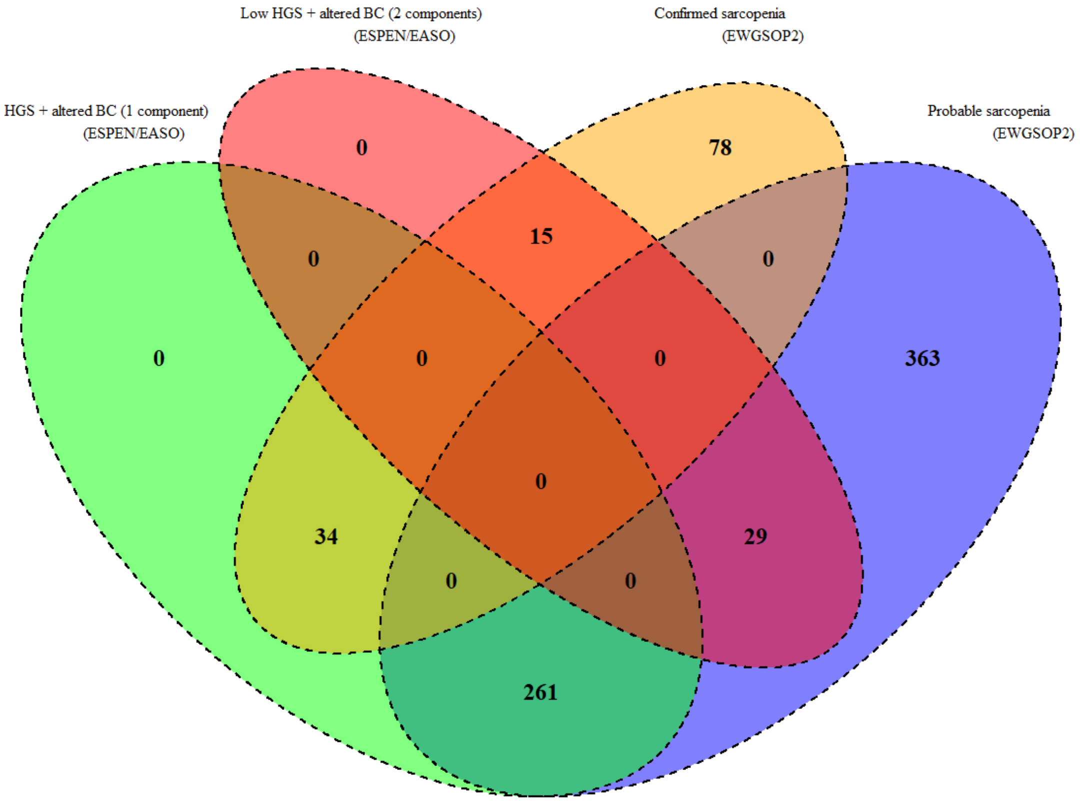

Abbreviations: HGS: handgrip strength, BC: body composition.

© 2024 Benz E et al. *JAMA Network Open.*

eReferences

1. Gallagher D, Heymsfield SB, Heo M, Jebb SA, Murgatroyd PR, Sakamoto Y. Healthy percentage body fat ranges: an approach for developing guidelines based on body mass index. Am J Clin Nutr. 2000;72(3):694-701.

2. Cruz-Jentoft AJ, Bahat G, Bauer J, Boirie Y, Bruyère O, Cederholm T, et al. Sarcopenia: revised European consensus on definition and diagnosis. Age and ageing. 2019;48(1):16-31.

3. Donini LM, Busetto L, Bischoff SC, Cederholm T, Ballesteros-Pomar MD, Batsis JA, et al. Definition and Diagnostic Criteria for Sarcopenic Obesity: ESPEN and EASO Consensus Statement. Obes Facts. 2022;15(3):321-35.

4. Hu B, Yang XR, Xu Y, Sun YF, Sun C, Guo W, et al. Systemic immune-inflammation index predicts prognosis of patients after curative resection for hepatocellular carcinoma. Clin Cancer Res. 2014;20(23):6212-22.

5. Guerrero-Romero F, Simental-Mendía LE, González-Ortiz M, Martínez-Abundis E, Ramos-Zavala MG, Hernández-González SO, et al. The product of triglycerides and glucose, a simple measure of insulin sensitivity. Comparison with the euglycemic-hyperinsulinemic clamp. J Clin Endocrinol Metab. 2010;95(7):3347-51.

© 2024 Benz E et al. *JAMA Network Open.*
