# Supplementary material for: Sarcopenia and Sarcopenic Obesity and Mortality Among Older People
Source: JAMA Netw Open. 2024 Mar 25;7(3):e243604. doi: 10.1001/jamanetworkopen.2024.3604 (PMC10964118; doi:10.1001/jamanetworkopen.2024.3604)
Supplement: Supplement 2. — Data Sharing Statement [file jamanetwopen-e243604-s002.pdf]

Data Sharing Statement

Benz. Sarcopenia and Sarcopenic Obesity and Mortality Among Older People. *JAMA Netw Open*. Published March 25, 2024. doi:10.1001/jamanetworkopen.2024.3604

Data
Data available: No
